# Huriez Syndrome and SCC Risk: A Narrative Review Highlighting Surgical Challenges and Oncologic Considerations

**DOI:** 10.3390/jcm14155214

**Published:** 2025-07-23

**Authors:** Alessia Pagnotta, Luca Patanè, Carmine Zoccali, Francesco Saverio Loria, Federico Lo Torto, Diego Ribuffo

**Affiliations:** 1 Hand and Microsurgery Unit, Jewish Hospital, 00148 Rome, Italy; pagale@me.com; 2 Plastic Surgery Unit, Department of Surgery, Sapienza University of Rome, 00185 Rome, Italy; 3 Orthopaedic Surgery Unit, Department of Surgery, Sapienza University of Rome, 00185 Rome, Italy

**Keywords:** Huriez syndrome, squamous cell carcinoma, genodermatoses, skin cancer, rare diseases

## Abstract

**Background**: Huriez syndrome is a rare hereditary skin disorder marked by early-onset sclerodactyly, hyperkeratosis of the palms and soles, and nail dysplasia. A key concern is the early and aggressive development of cutaneous squamous cell carcinoma (SCC), typically affecting the dorsal aspects of the hands. **Methods**: This narrative review summarizes clinical features, genetic aspects, and oncologic implications of Huriez syndrome. A systematic search was conducted in PubMed and Scopus, including English-language articles published up to May 2025. Relevant case reports and small case series were analyzed. **Results**: Seven patients (58.3%) underwent multiple surgeries due to recurrent or bilateral disease. Six patients (50%) required amputations, including finger, hand, and arm amputations, with no foot amputations reported. Reconstruction after oncological resection was performed in six patients (50%) using skin grafts (3), pedicled flaps (2), or free flaps (1). Amputation was mainly for advanced disease, with radial forearm flaps used for reconstruction. All flaps remained disease-free. Five cases (41.6%) had a history of local recurrence. **Conclusions:** The early diagnosis of Huriez syndrome is crucial to enable the surveillance and timely treatment of SCC. A multidisciplinary team including dermatologists, oncologists, plastic surgeons, and geneticists is recommended. Further research is needed to clarify genetic mechanisms and develop early detection strategies to improve outcomes.

## 1. Introduction

Squamous cell carcinoma (SCC) is the most prevalent form of primary malignant tumor found in the hand [1]. SCC arises from the malignant transformation of keratinocytes and shows varying levels of aggressiveness, influenced by factors like tumor differentiation, anatomical site, immune system status, and delay in diagnosis. It is significantly more common in the hand than basal cell carcinoma (BCC), with a BCC to SCC ratio of 1:14 [2]. Unlike BCC, SCC has a higher potential for local invasion, bone involvement, and lymphatic metastasis, necessitating early detection and intervention. SCC progresses from precursor lesions like actinic keratosis to carcinoma in situ (Bowen’s disease) and eventually to invasive SCC [3].

The development of SCC is primarily driven by chronic ultraviolet (UV) radiation exposure, especially in sun-exposed areas like the dorsal hands. Other contributing factors include carcinogenic agents such as arsenic, polycyclic hydrocarbons, and Human Papilloma Virus (HPV) infections. Additionally, individuals with genetic conditions like xeroderma pigmentosum and Huriez syndrome and those with chronically inflamed or damaged skin, are at higher risk for SCC development.

**Figure 1:**
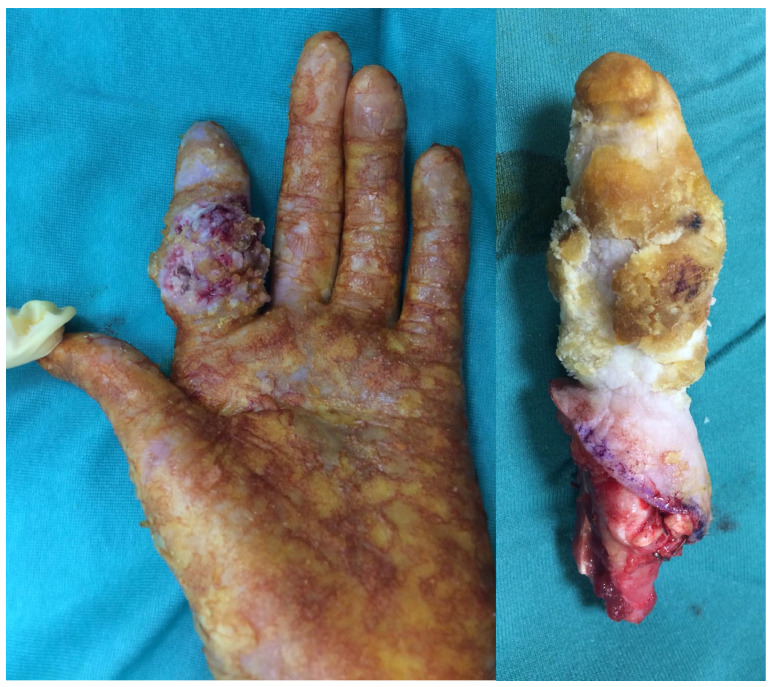
A patient with Huriez syndrome and a cutaneous SCC of the second finger.

Huriez syndrome, also known as sclerotylosis, is a rare autosomal dominant genodermatosis characterized by congenital scleroatrophy of the distal extremities, palmoplantar keratoderma, and nail hypoplasia [4,5]. First described in 1963 in two French families, it has since been reported in fewer than 50 cases worldwide, making it one of the rarest cancer-prone genodermatoses [6,7,8]. A hallmark of this condition is the development of aggressive cutaneous squamous cell carcinoma (SCC), particularly on the dorsal aspect of the hands, often at an early age (Figure 1). Unlike SCCs associated with chronic sun exposure or immunosuppression, those arising in Huriez syndrome may follow a more rapid clinical course and are more prone to local recurrence or regional lymph node metastasis [9,10], necessitating wide local excisions or even amputations, similar to what is needed for other soft tissue tumors of the hand [11].

Reports suggest that up to 15–20% of affected individuals develop SCC, usually before the age of 45, which underscores the malignant potential of this syndrome [12]. Clinically, the syndrome often begins at birth or early infancy with signs of scleroatrophy, tightly adherent skin on the hands and feet, and progressive keratoderma. Nail hypoplasia may develop during childhood, often leading to significant nail plate dystrophy or anonychia. Some patients may also exhibit features of poikiloderma, hypopigmentation, or sweat gland dysfunction [13,14]. Although its exact genetic basis remains unclear, recent studies have identified a possible link to pathogenic variants in the SMARCAD1 gene, which encodes a chromatin remodeling helicase with a role in maintaining skin barrier integrity [15]. These findings have expanded the spectrum of genodermatoses associated with chromatin remodeling defects and may explain the high susceptibility to early oncogenesis observed in these patients. Due to its rarity, there are currently no standardized surveillance protocols or management guidelines specific to Huriez syndrome. While small, low-risk lesions of hand SCC are commonly treated with shave excision, curettage, cryotherapy, or Mohs micrographic surgery, SCC in Huriez patients is characterized by earlier onset, more aggressive behavior, and a higher incidence of metastasis, making it unsuitable for standard low-risk management.

Nevertheless, delay or avoidance of biopsy is frequently observed due to the atypical presentation and chronicity of skin changes, which, in turn, contributes to the risk of late-stage SCC diagnosis. In the absence of guidelines, the early identification of clinical signs, regular dermatologic follow-up, and a low threshold for biopsy of suspicious lesions are crucial in preventing advanced-stage disease [16,17]. Despite the recognition of the syndrome’s increased risk for aggressive SCC, there are only a few reports in the literature regarding its surgical management and treatment protocols. This lack of comprehensive data underscores the absence of standardized guidelines for the management of Huriez syndrome, especially in terms of early diagnosis, treatment strategies, and post-operative care. The aim of this narrative review is to consolidate the available clinical evidence on Huriez syndrome, with a particular focus on the management of SCC in this rare and complex condition. In addition to synthesizing the existing literature, we integrate our own clinical experience in managing Huriez syndrome, emphasizing key treatment pearls that should be considered when treating patients with this disease. By combining evidence from the literature with our practical insights, we highlight critical factors for successful management, offering valuable guidance for clinicians dealing with the challenges posed by Huriez syndrome and its associated complications.

## 2. Materials and Methods

### 2.1. Search Strategy and Study Selection

For this narrative review, a comprehensive search was conducted across electronic databases, including MEDLINE, Pubmed, Pubmed Central (PMC), and the Cochrane Library, targeting clinical studies related to Huriez syndrome. The search strategy utilized Medical Subject Headings (MeSHs) and specific keywords: “Huriez syndrome [MeSH]” and sclerotylosis [MeSH]”. This narrative review was conducted with attention to methodological quality, following the guidance outlined in the Scale for the Assessment of Narrative Review Articles (SANRA) and referring to relevant aspects of the PRISMA-ScR checklist, though not conducted as a formal scoping review as defined by PRISMA-ScR [18].

Only articles published in English were considered. Eligible studies included case reports, small case series, and narrative or pictorial reviews with detailed clinical descriptions and histopathological confirmation. Articles lacking clinical data, not directly related to Huriez syndrome, or without confirmed SCC were excluded. Titles and abstracts were screened first, followed by full-text analysis of relevant records.

### 2.2. Data Extraction and Analysis

Extracted data included patient demographics, clinical phenotype, age at diagnosis, family history, the presence and characteristics of SCC, localization, histopathological findings, treatment approaches, and follow-up. Special focus was given to features of malignant transformation, recurrence patterns, and reconstructive strategies.

Data extraction was conducted independently by two authors (AP and DR). Disa-greements on study eligibility were resolved through consensus discussions with the other authors. Reference lists from the retrieved articles were examined to ensure that a comprehensive literature search was performed.

Given the rarity of Huriez syndrome and variability in published cases, statistical synthesis was not feasible. The results are, therefore, presented as a qualitative descriptive synthesis, aimed at identifying consistent patterns, highlighting diagnostic challenges, and outlining areas for future research.

## 3. Results

**Figure 2:**
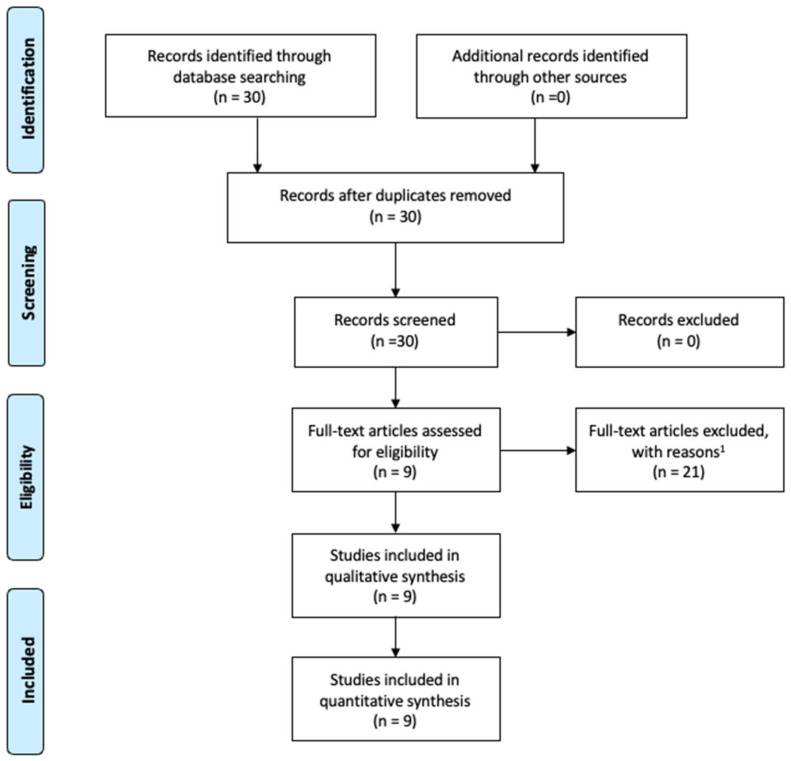
PRISMA flow diagram. ^(1)^ Not in English, letter, book section, experimental, animal studies.

A total of 30 studies were identified in the initial search. After screening titles and abstracts, nine papers on Huriez syndrome-related SCC were included in the final review. The full texts and references were carefully reviewed to identify additional relevant studies (Figure 2).

### 3.1. Demographic and Clinical Characteristics

A total of 9 studies published between 1995 and 2024 were included, reporting on 12 patients diagnosed with Huriez syndrome complicated by cutaneous SCC [8,19,20,21,22,23,24,25,26]. Of these, seven were male (58.3%) and five were female (41.7%). The average age at SCC diagnosis was 46.7 years (±11.6), with a median of 43 years (range: 22–62) (Table 1).

### 3.2. Tumor Localization and Laterality

All 12 patients (100%) developed SCC in the hands, with 3 cases (25%) also involving the feet. Among those with hand involvement, three patients (25%) had bilateral disease, whereas nine (75%) were unilateral. The palmar and volar surfaces were frequently involved, with recurrent tumors often requiring more extensive resections.

### 3.3. Surgical Management

Seven patients (58.3%) underwent multiple surgeries due to recurrent, bilateral disease or hand/foot involvement. Six (50%) patients underwent amputations. The amputation of fingers, hand, and arm is described in four papers. Five patients (41.6%) had multiple and bilateral finger amputations. No foot amputations were described in Huriez patients. Six patients (50%), after oncological resection, underwent reconstruction with skin grafts (3), pedicled flaps (2), or free flaps (1).

**Figure 3:**
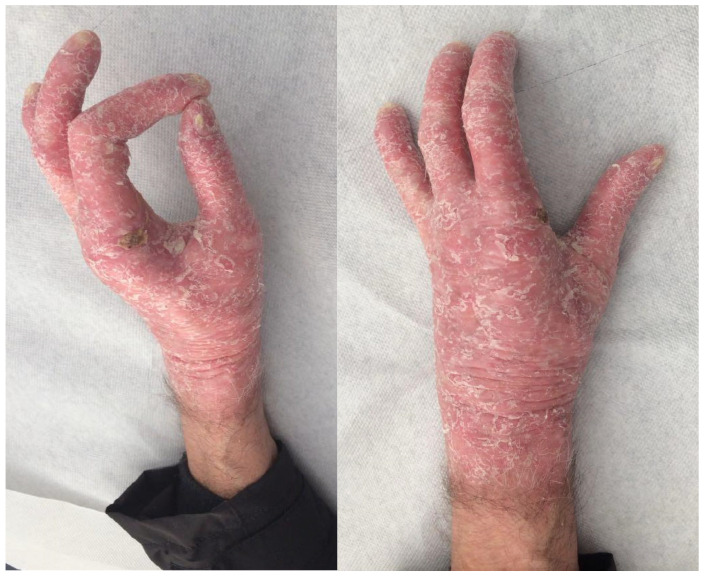
The same patient as in Figure 1, following the second finger amputation. The patient healed well with optimal functional recovery.

1. Amputation. Cases requiring finger amputation had locally advanced disease or recurrence. The index was the most affected finger (50%) (Figure 3). The palmo-volar skin was the area affected in all cases. Two patients had wrist and arm amputation. The wrist amputation was decided in a patient who had had six local recurrences. The patient with arm amputation died from lung metastatis.
2. Skin grafts. Five cases were treated with excision and skin grafts. In three cases, revision surgery was necessary for recurrences or inadequate margins.
3. Skin flaps, pedicled and free. Two patients were treated with radial forearm flap for third ray reconstruction, after second ray amputation [24,26]. The flap was harvested from the forearm, where the skin was unaffected by scleroatrophy. In both cases, the skin of the flaps at the recipient site remained free of the disease. One patient had SCC in the right foot and was treated with a free radial forearm flap harvested from the amputated arm.

### 3.4. Recurrence and Histological Grading

Data for local recurrence were present in five of the nine papers. Five cases (41.6%) experienced local recurrences, including a case with six documented relapses prior to wrist amputation. Histologically, 67% of tumors were classified as Grade 1 (well-differentiated), 13% were classified as Grade 2 (moderately differentiated), and 20% were reported as carcinoma in situ. Recurrences were often linked to incomplete surgical margins and delays in initial biopsy.

### 3.5. Metastasis and Outcomes

Metastatic spread occurred in four patients (33.3%), primarily involving lymph nodes and lungs, who subsequently died from metastatic disease. The patient described by Guerriero was 60 years old, with hand and foot localization and sustained arm amputation. Three patients described before the 2000s by Kavanach, Hamm and Delaporte died from metastatic disease. The patient described by Delaporte was 35 years old and sustained multiple recurrences in both hands. The younger patient, aged 22 years old, as reported by the sister of the patient, was the only female patient who died from disease, and her medical history was briefly described in the paper of Kavanach.

## 4. Discussion

### 4.1. Clinical and Oncologic Implications of SCC in Huriez Syndrome

Huriez syndrome is recognized as a cancer-prone genodermatosis due to its strong association with cutaneous SCC, particularly in the acral regions. Unfortunately, the development of SCC remains the most important prognostic element for these patients. The review of the available literature confirms that SCC in these patients tends to occur at a relatively young age, with a median of 43 years. All reported cases involved the hands, and 25% also showed foot involvement, highlighting the typical acral predilection of tumor development in this syndrome. The aggressive nature of SCC in Huriez syndrome, with early onset and potential for multifocality, reinforces the need for heightened clinical suspicion and prompt histological confirmation of suspicious lesions. The chronic scleroatrophy and keratoderma can obscure early signs of malignancy, contributing to diagnostic delays.

### 4.2. Recurrence and Metastatic Risk

Local recurrence was reported in 41.6% of cases, often associated with incomplete surgical margins and delayed biopsies. One patient experienced six local recurrences prior to undergoing wrist amputation, highlighting the critical importance of adequate initial resection, similar to what is needed for other soft tissue tumors of the hand [27,28,29,30,31,32,33]. Histologically, the majority of tumors were well-differentiated (67%), but moderately differentiated and in situ lesions were also observed. While histologic grading can provide insights into tumor behavior, it does not fully predict recurrence or metastatic potential in these patients. Notably, metastasis to regional lymph nodes or lungs occurred in 33.3% of cases, with a total of four patients succumbing to the disease. This included a 22-year-old woman, illustrating that even young patients are not spared from aggressive tumor progression.

### 4.3. Surgical Management and Reconstructive Challenges

The management of cutaneous SCC in Huriez syndrome presents unique challenges and often requires multiple surgical procedures. In the cohort analyzed, 58.3% of patients underwent more than one operation due to recurrences or multifocal disease.

Delayed diagnoses not only complicate treatment but also compromise hand dexterity and prehension following demolitive surgery, emphasizing the importance of early recognition and management. The treatment of SCC in Huriez syndrome generally involves oncological surgical excision, but several factors complicate management. First, rapid tumor growth is common, with early invasion into tendons, neurovascular bundles, and bone tissues, making complete resection particularly challenging. Second, the presence of scleroatrophy in the peritumoral skin makes it difficult to achieve clear surgical margins, as the affected skin often appears suspicious, increasing the risk of incomplete excision and local recurrence. In planning resection, margins should be wider than 1 cm in the presence of suspicious areas [24], because incomplete excision is a major cause of local recurrences and amputation.

Third, local flaps, which are commonly used in hand reconstruction, are often impractical due to the high likelihood of additional precancerous or cancerous lesions in surrounding tissue, further complicating the reconstruction process. In cases of significant tissue loss involving the exposure of bone, tendons, and neurovascular pedicles, the radial forearm flap proved to be the optimal choice, as it is harvested from an unaffected area. As highlighted in our previous study [26], flaps play a crucial role in disease management, as the skin grafts remain unaffected by the disease, while the surrounding skin benefits from the flap placement, leading to a regression of scleroatrophy. Consistent with the recommendations of other authors [22,24], we believe that preventive excision with grafts or flaps could be considered for these patients, provided it is supported by a multidisciplinary team evaluation.

These factors necessitate a comprehensive, multidisciplinary approach to treatment. We believe that careful planning and management are essential for optimal outcomes in these patients. A thorough evaluation by a multidisciplinary team, including dermatologists, plastic surgeons, and oncologists, is crucial to address the complexities of this condition.

### 4.4. Surveillance and Multidisciplinary Strategy

Given the rarity and severity of Huriez syndrome, there are no standardized surveillance protocols. The small number of published studies and limited cohort sizes significantly restrict the ability to generalize conclusions or formulate definitive treatment guidelines. Furthermore, most studies lack long-term follow-up data, leaving long-term outcomes and potential complications poorly understood. These limitations underscore the need for further research, including larger studies with extended follow-up periods, to develop more comprehensive management strategies and improve clinical outcomes for patients with Huriez syndrome. However, the available data support the need for proactive monitoring and early intervention. Regular dermatologic evaluations and a low threshold for performing biopsies are recommended, particularly when lesions exhibit changes in texture, color, or growth. In many cases, delays in biopsy were associated with advanced disease at diagnosis.

We want to highlight that biopsy is the first step of the treatment to confirm diagnosis and grading. According to the INCa-HAS recommendations of 2009, titled “Diagnostic and therapeutic management of cutaneous squamous cell carcinoma”, the excision of the infiltrating cSCC should have margins of one centimeter [34]. A multidisciplinary approach involving dermatologists, oncologists, plastic surgeons, and geneticists is essential for optimal care. Genetic counseling should also be considered, given the hereditary nature of the syndrome and emerging evidence linking it to SMARCAD1 mutations.

### 4.5. Future Directions

Given the rarity of Huriez syndrome, collaborative efforts are essential to expand clinical knowledge and improve management. Key areas for future research include multicenter registries to better define incidence, clinical variability, and prognosis as well as shared clinical guidelines to standardize surveillance and early intervention for suspicious lesions.

Using advanced imaging techniques, possibly supported by artificial intelligence, to enhance early SCC detection may increase patient survival or avoid ample resections in the future. Currently, there is no definitive evidence to suggest that performing a lymph-node biopsy improves prognosis in these patients, and future research is needed to clarify if patients will benefit from this practice [35,36,37,38,39,40,41].

## 5. Conclusions

Huriez syndrome represents an exceptionally rare genodermatosis with a high risk of developing early-onset and aggressive cutaneous squamous cell carcinoma, particularly on the hands. Despite the limited number of reported cases, a consistent pattern of clinical features and surgical challenges emerges, including frequent local recurrences, high rates of amputation, and a non-negligible risk of metastasis. Early biopsy is paramount for accurate diagnosis and staging, followed by the early and ample resection of the tumor to ensure adequate margins and reduce the risk of recurrence. Reconstruction with distant flaps, such as radial forearm flaps or free flaps, should be considered for coverage, as these techniques offer disease-free tissue for hand reconstruction, minimizing the risk of recurrence and providing functional outcomes. In our clinical experience, this approach ensures that the hand remains covered with healthy, non-sclerotic tissue, optimizing both oncologic and functional results.

## Figures and Tables

**Table 1:** The literature review.

Authors	N° Patients	Age	Gender	Localization	Histology	Surgery	Recurrence	FU	Metastasis
Patrignani et al. (2024) [26]	1	55	M	R Hand L Hand R Foot	G1 G1 G1 G1	Wrist amputation 2nd ray amputation Radial forearm flap Free radial forearm flap	6 1	5 y 3 y 2 y	___
Halbony et al. (2022) [25]	1	41	F	R Foot R Hand	In situ In situ	Ex, skin graft	___	2y	___
Dumont et al. (2013) [24]	1	62	M	L Hand	G1	Fingers amputation 2nd ray amputation Skin graft Radial forearm flap	2	1 y	___
Guerriero et al. (2008) [23]	1	60	M	R Hand R Foot		Arm amputation Ex	___	1 y	Lung
Riggio et al. (2005) [22]	1	46	M	L Hand R Hand	G1 G2 G1 G1	Ipothenar ex, skin graft Palmar ex, skin graft Ipothenar ex, skin graft Amputation 4th + 5th ray	1 1		N (G1)
Watanabe et al., 2003 [21]	1	41	F	L Hand R Hand	G1 In situ	Thumb amputation Thumb ex, skin graft	1y	___	___
Kavanagh et al. (1997) [20]	2	22 45	F F	Hand Hand	___	___	___	___	M
Hamm et al. (1996) [8]	1	37	F	R Hand	G2	Thenar			M (N + Lung)
Delaporte et al. (1995) [19]	3	35 55 39	M M M	Hands R Hand L Hand	G1	Fingers Thumb 2nd ray amputation	7		M
